# Crystallization of Isotactic Polypropylene Nanocomposites with Fibrillated Poly(tetrafluoroethylene) under Elevated Pressure

**DOI:** 10.3390/polym14010088

**Published:** 2021-12-27

**Authors:** Przemyslaw Sowinski, Sivanjineyulu Veluri, Ewa Piorkowska

**Affiliations:** Centre of Molecular and Macromolecular Studies, Polish Academy of Sciences, Sienkiewicza 112, 90-363 Lodz, Poland; sveluri@cbmm.lodz.pl

**Keywords:** crystallization, gamma form, poly(tetrafluoroethylene) fibers, isotactic polypropylene, high pressure

## Abstract

Nanocomposites of isotactic polypropylene with 1–5 wt.% of fibrillated PTFE (PP/T) were prepared, and their crystallization during cooling under elevated pressure, in a wide pressure range, up to 300 MPa, as well as the resulting structure, were examined. The crystallization peak temperatures of PP/T, especially with 3 and 5 wt.% of PTFE, exceeded by up to 13 °C those of neat PP. Moreover, a fine-grain structure was formed in PP/T in the entire pressure range, which proved the ability of the fibrillated PTFE to nucleate crystallization of PP in the γ-form under elevated pressure. This also resulted in a higher crystallinity level developed in the γ-domain, before the temperature range of the α-domain was reached during cooling. Hence, the γ-content increased in comparison to that in neat PP, under the pressure up to 200 MPa, especially under 50–100 MPa.

## 1. Introduction

All-polymer composites, including self-reinforced composites, are an interesting group of materials. The advantages of these materials include relatively low weight; easy recycling ability, e.g., reprocessing by remelting; and the possibility of tuning adhesion between the components [1,2]. Such composites can be obtained by filling polymers with ready-made fibers or by the formation of fibers in a matrix, in situ during processing [2]. Composites, in which polymer particles with a reduced density of entanglements undergo deformation and fibrillation during processing, belong to the second group [3,4,5,6]. For instance, poly(tetrafluoroethylene) (PTFE) nanofibrils were formed during the shearing of PTFE powder particles with reduced entanglement density during compounding with melts of other polymers, including isotactic polypropylene (iPP), high density polyethylene (HDPE), low density polyethylene, poly(oxymethylene), and atactic polystyrene [3,4,5,7]. Composites with PTFE nanofibrils exhibited improved modulus of elasticity, yield stress and strain at break during tensile drawing, as well as strain hardening during the extensional deformation of the melt [3,5,8,9]. The enhancement of the clarity of iPP and HDPE nanocomposites with PTFE nanofibers was also reported [4]. It was observed that PTFE nanofibers strongly nucleated the crystallization of iPP [4,10,11], leading to the formation of transcrystalline zones around the fibers, an increase of nonisothermal crystallization temperature, and a decrease of crystallization half-time during isothermal crystallization. It is worth mentioning that PTFE particles also strongly nucleated the crystallization of iPP [12].

iPP can crystallize in different crystallographic forms. The most common, the monoclinic α-form, exhibits characteristic crystallographic branching, resulting in ‘cross-hatched’ lamellar morphology, with a ‘daughter’ lamellae tilted at an angle of ca. 80° or 100° to a ‘mother’ lamellae. In turn, the crystallization of iPP in the triclinic β-form requires specific conditions of crystallization, such as zone solidification [13], or the use of special nucleating agents [14,15]. The orthorhombic γ-form was found in low molar mass iPP [16,17,18], in propylene copolymers with a small amount of 1-olefine co-units [19,20,21,22], and in iPP with stereo- or regio-defects [23,24,25]. Moreover, the crystallization of iPP in the γ-form is facilitated by high pressure [26,27,28,29]. In addition, copolymers of propylene with more than 10% of hexene or pentene comonomers crystallized in the trigonal form δ [30,31]; whereas in stereo-defective iPP the orthorhombic ε-form was discovered [32]. In turn, at very high undercoolings the formation of smectic meso-phase was observed [33,34].

The γ-form is unique because of a peculiar arrangement of iPP chains in the crystal. The γ-lamellae are composed of successive bi-layers with the chain axes parallel, but inclined by ca. 80° to those in the adjacent bi-layers, being tilted by ca. 40° with respect to the lamellar normal [35,36,37]. Although the γ-phase was detected in nucleated iPP crystallized under atmospheric pressure [38], the crystallization of the γ-form is facilitated by elevated pressure, as mentioned above. According to the phase diagram developed by Mezghani and Phillips [29], the crystallization in the γ-form occurs at high temperature. With increasing pressure, the temperature range of γ-phase formation broadens. The temperature above which the γ-modification forms, weakly depends on pressure, in comparison to the equilibrium melting temperature, T_m_^0^, of the γ-form, which increases from 186.5 ℃ at atmospheric pressure, to 241.1 °C at 200 MPa [29]. It is worth mentioning that γ-iPP crystallized under high pressure can exhibit higher modulus of elasticity and yield stress than α-iPP [39,40].

Both, PTFE fibers and particles nucleate crystallization of iPP in the α-form under atmospheric pressure [3,12]. However, PTFE particles also efficiently nucleate the crystallization of iPP under high pressure in the orthorhombic γ-form, which was reflected in the increase of crystallization temperature and the reduction of grain-size of iPP [41,42,43].

The influence of elevated pressure on crystallization is of interest because of injection molding. Our study focused on nonisothermal crystallization of iPP with fibrillated PTFE during cooling, in a broad pressure range, up to 300 MPa. PTFE fibrils formed during the mixing of PTFE powder with molten iPP. The effect of these nanofibers on the crystallization temperature and structure of the nanocomposites was determined. The structure of the nanocomposites was analyzed using scanning electron microscopy (SEM) and polarized light microscopy (PLM), as well as by wide angle X-ray diffraction (WAXD) and differential scanning calorimetry (DSC). It was found that the PTFE fibrils efficiently nucleated crystallization in the γ-form, altering the structure of the iPP and increasing the crystallization temperature and also the γ-content, the latter especially in the pressure range of 50–100 MPa.

## 2. Materials and Methods

### 2.1. Materials

iPP Adstif HA740N (PP) with a melt flow rate (MFR) of 12 g/10 min (230 °C/2.16 kg) and density of 0.9 g/cm^3^ was provided by Basell Orlen Polyolefins (Plock, Poland). Anox 20 and Ultranox 626 were supplied by Addivant (Danbury, CT, USA). An acrylic-modified poly(tetrafluoroethylene) Metablen A 3000 (PTFE) from Mitsubishi Rayon Carbon Fiber and Composites (Irvine, CA, USA) was used to modify PP. Prior to further use, the PTFE powder was sifted through a 0.2 mm sieve.

Nanocomposites of PP with 1, 3, and 5 wt.% of PTFE (PP/T1, PP/T3, and PP/T5) were prepared by compounding in a Brabender (Duisburg, Germany) batch mixer at 195 °C. During the first 6 min PP was mixed with 0.2 wt.% of Anox 20, 0.1 wt.% of Ultranox 626, and 0.2 wt.% of calcium stearate. Next, PTFE powder was added, and over 4 min the rotation speed was increased to 100 rpm, and the mixing continued for another 10 min. Neat PP was processed under the same conditions, to obtain a reference material.

### 2.2. Crystallization under High Pressure

Specimens of approximately 200 mg of the materials, in the form of disks 9.5 mm in diameter, were placed in a custom-built cell consisting of a barrel and pistons, equipped with heaters and a temperature sensor. Detailed description of the cell was given elsewhere [41,42,43,44,45]. To pressurize the specimens, an Instron tensile testing machine (Instron Corp., High Wycombe, UK) compressed the pistons along the cell axis, at a cross-head speed of 2 mm/min. The hydrostatic pressure and temperature in the cell were controlled, with an accuracy of ±0.5 MPa and 1 °C, respectively.

**Figure 1:**
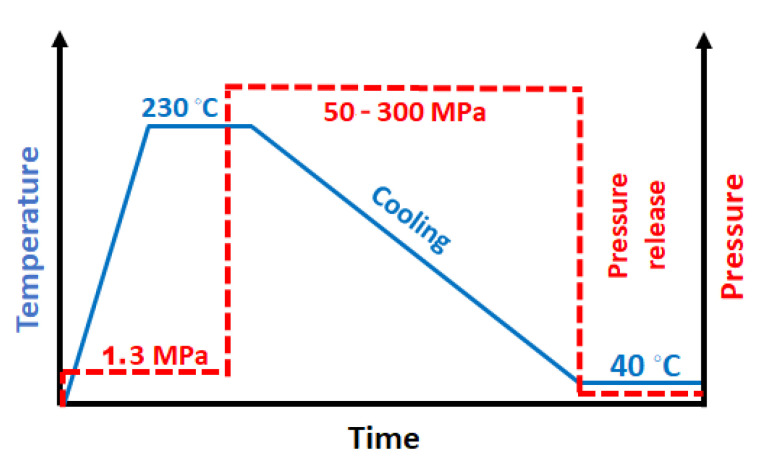
Pressure and temperature protocol.

The pressure and temperature protocol is schematically shown in Figure 1. First, the specimens were heated to 230 °C under a pressure of 1.3 MPa, which was applied to ensure good thermal contacts in the cell. The specimens were held at 230 °C for 3 min, to erase their thermal history, and then the pressure was increased to 50, 100, 200, or 300 MPa. Then, the specimens were cooled under pressure to 40 °C, and the pressure was released. Crystallization during cooling under 1.3 MPa was also conducted for comparison. After the crystallization, the specimens were analyzed with WAXD, PLM, and DSC. The cooling rate was not controlled, but it was reproducible and nearly constant, close to 8 °C/min, in the temperature range of PP crystallization.

During cooling, the decrease of volume due to crystallization was monitored by measuring the cross-head displacement. The cross-head displacement rate exhibited a peak corresponding to the peak of crystallization rate. The simultaneous measurement of the temperature allowed determining the temperature of the peak of the cross-head displacement rate, which was taken as the crystallization temperature, T_c_, as previously described [42].

### 2.3. Characterization

The structure of the nanocomposites was studied by SEM. To reveal their internal structure, PP and PP/PT nanocomposites were cryo-fractured. In addition, to dissolve the PP matrix and better expose the fibrillated PTFE, the materials were treated with xylene (Chempur, Poland), at its boiling temperature of 139 °C. The specimen surfaces were sputtered with gold using an Edward Sputter Coater (Crawley, UK) and examined by SEM JEOL JSM-5500LV (Tokyo, Japan) operating in SEI high vacuum mode at an accelerating voltage of 10 kV.

The crystallographic forms and crystallinity degree (X_c_) in the crystallized specimens were analyzed by WAXD, in the reflection mode, using a Panalytical Xpert’ PRO diffraction system from Malvern Panalytical Ltd. (Malvern, UK), with CuKα radiation (0.154056 nm), operating at 40 kV and 30 mA. The diffractograms were recorded in the 2θ range, from 10° to 70°, and deconvoluted using WAXSFIT 4.0 program [46] (ATH, Bielsko-Biala, Poland), as previously described [41,42,43,45]. The contents of the α- and γ-modifications in the crystalline phase, K_α_ and K_γ_, were determined according to the method proposed by Turner-Jones et al. [47], based on the formula:

(1) $$K_{\gamma }=I{\left(117\right)}_{\gamma }/{\left[I{\left(117\right)}_{\gamma }+I{\left(130\right)}_{\alpha }\right]}^{-1}$$

(2) $$K_{\alpha }=1-K_{\gamma }$$

where I denotes the integral intensity of the respective peak.

The amorphous halos were also determined by the deconvolution, and X_c_ values were calculated.

To study the semicrystalline morphology, 10-µm thick sections of the materials were microtomed and examined by means of PLM (PZO, Warsaw, Poland) equipped with a video camera.

To study the crystallization and melting of the materials under atmospheric pressure (P_atm_), a DSC 2920 from TA Instruments (New Castle, DE, USA) was used. Approx. 6–8 mg specimens were analyzed. To examine the crystallization under P_atm_, the specimens were heated up at 10 °C/min to 230 °C, and after holding for 3 min at this temperature, cooled down at 10 °C/min. The melting of the specimens crystallized under various pressures was studied during heating at 10 °C/min to 230 °C.

## 3. Results and Discussion

**Figure 2:**
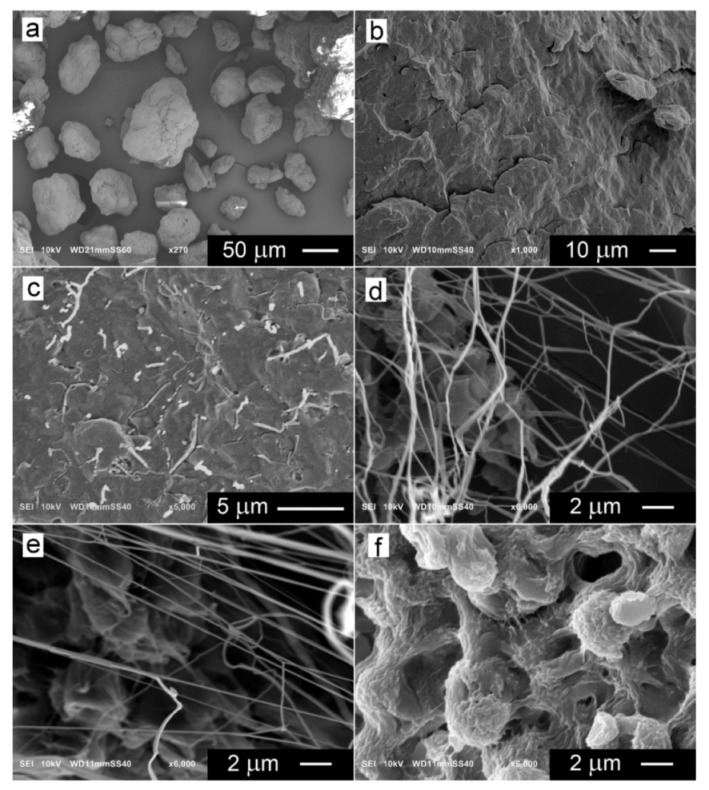
SEM micrographs of PTFE powder (**a**), cryo-fracture surface of neat PP (**b**), and PP/T5 (**c**), PTFE fibrils exposed by dissolution of PP/T5 (**d**) and PP/T1 (**e**), neat PP after treatment with boiling xylene (**f**).

SEM examination of the PP/T nanocomposites confirmed that fibrillation of the PTFE particles occurred during processing. Figure 2a shows the PTFE powder. Figure 2b,c compares the SEM micrographs of cryo-fracture surfaces of neat PP and PP/T5, respectively. In the latter, thin PTFE fibrils are visible. Only the fibril ends are seen because the fibrils were broken during fracture. Dissolving of the PP matrix in boiling xylene allowed better exposure of the PTFE fibrils, as shown in Figure 2d,e; mostly with diameters close to 200 nm, although occasionally thicker ones were also found. The thickest fibrils seen were, in fact, bundles of the thinner ones, possibly agglomerated during the dissolving of the matrix polymer. Neither single powder particles nor their agglomerates were found in the SEM micrographs of PP/T nanocomposites. The particles seen below the fibrils are remnants of PP, which did not dissolve in xylene. SEM micrograph of neat PP treated with xylene is shown in Figure 2f, for comparison.

**Figure 3:**
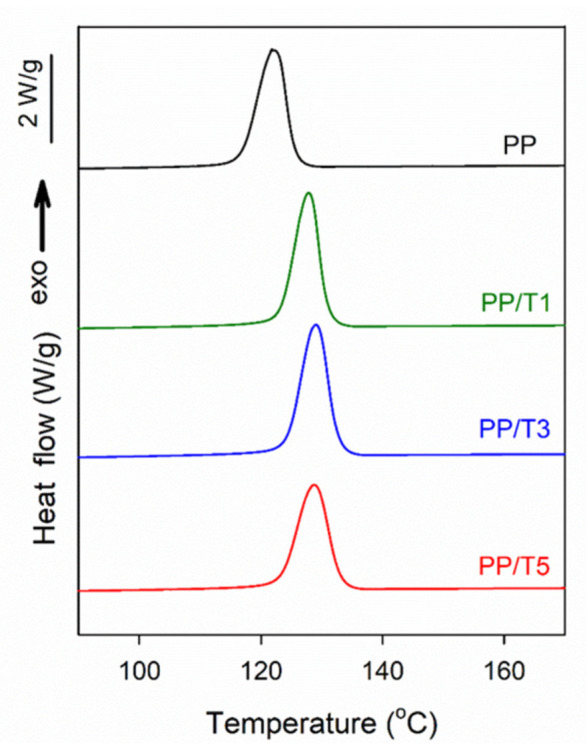
DSC cooling thermograms of PP and PP/T nanocomposites recorded at 10 °C/min.

DSC cooling thermograms of neat PP and PP/T are shown in Figure 3. The presence of fibers increased the crystallization peak temperature (T_c_) of the PP matrix, from 123 °C to 127, 128, and 129 °C, for PP/T1, PP/T3, and PP/T5, respectively. The crystallization enthalpy of the materials was approximately 100 J/g. The increase of T_c_ evidenced the nucleation activity of the PTFE fibrils in PP under P_atm_.

**Figure 4:**
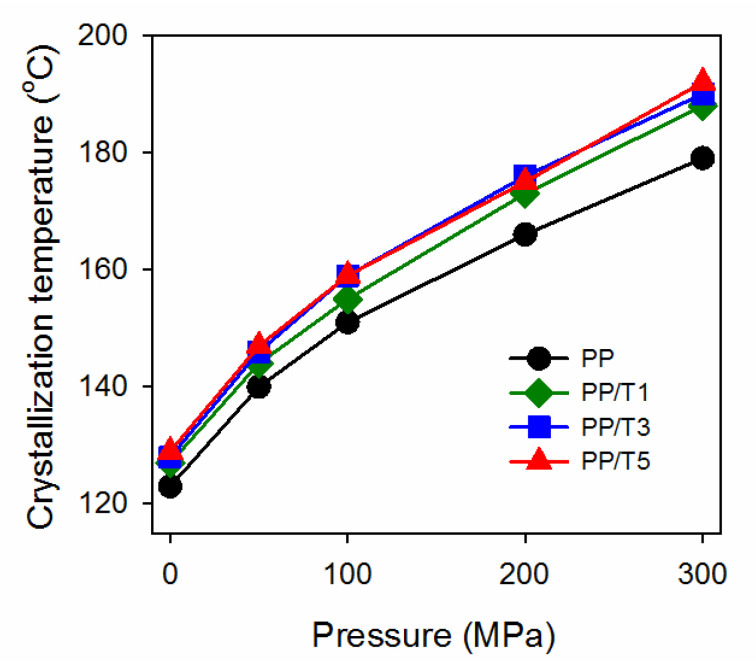
Crystallization temperatures, T_c_, of neat PP and PP/T nanocomposites crystallized during cooling under various pressures.

**Figure 5:**
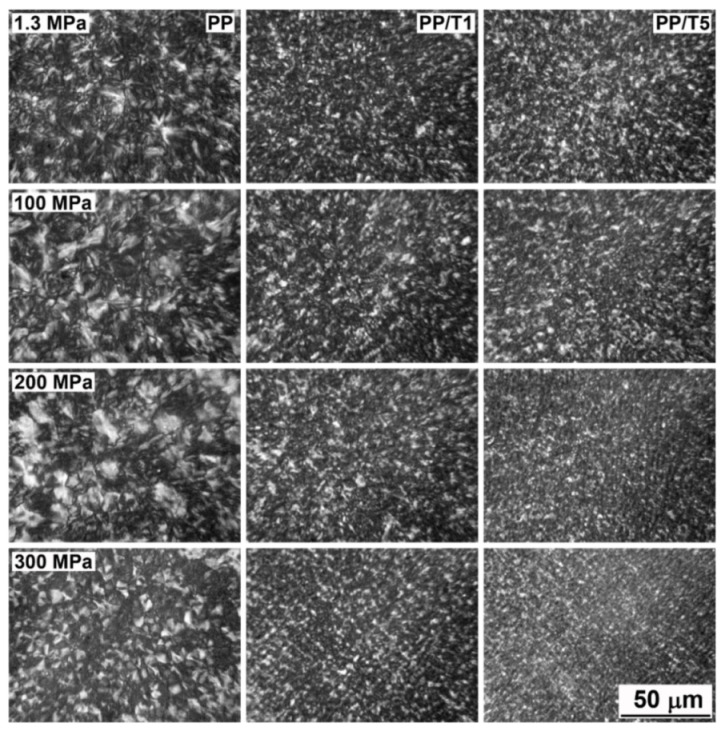
PLM micrographs of thin sections PP, PP/T1, and PP/T5 crystallized during cooling under various pressures.

Figure 4 shows the dependence of the T_c_ of neat PP and PP/T on crystallization pressure. It was not possible to measure the T_c_ of the materials under 1.3 MPa by monitoring the cross-head displacement; thus, the crystallization peak temperatures during cooling in DSC are plotted instead. Figure 4 shows that the T_c_ values of all materials increased with increasing pressure, as was expected, as the phase transition temperatures of PP depend on pressure [29]. The T_c_ of neat PP ranged from 123 °C at P_atm_ to 179 °C at 300 MPa. However, the T_c_ values of the PP/T exceeded those of neat PP in the entire pressure range, rising from 127–129 °C at P_atm_, to 188–192 °C at 300 MPa, and were the lowest and the highest for PP/T1 and PP/T5, respectively. The increase of T_c_ of PP/T evidenced the nucleation activity of the PTFE fibrils, which was also confirmed by the PLM analysis of morphology. PLM micrographs of PP, PP/T1, and PP/T5 are compared in Figure 5. In neat PP, spherulites with diameters up to 20–25 µm are visible, whereas in PP/T, the grain sizes were much smaller, regardless of the crystallization pressure, due to numerous nucleation sites. In addition, in many places the morphology of PP/T was suggestive of fibrillar nucleation sites.

**Figure 6:**
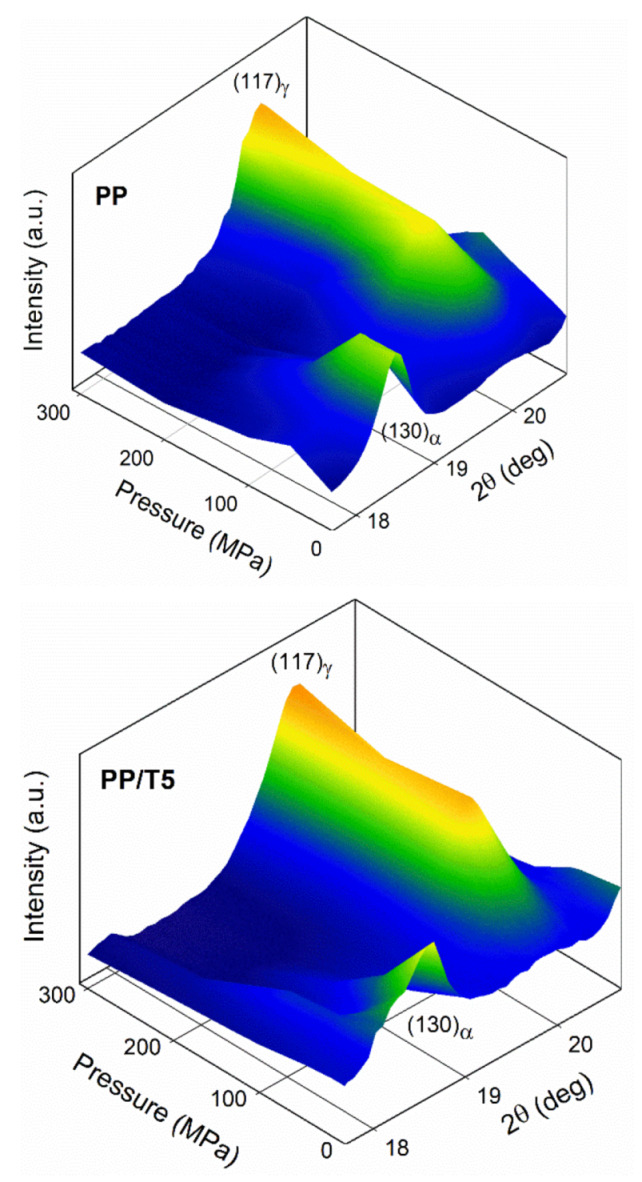
(130)_α_ and (117)_γ_ peaks **in** WAXD diffractograms of PP and PP/T5 crystallized during cooling under various pressures.

**Figure 7:**
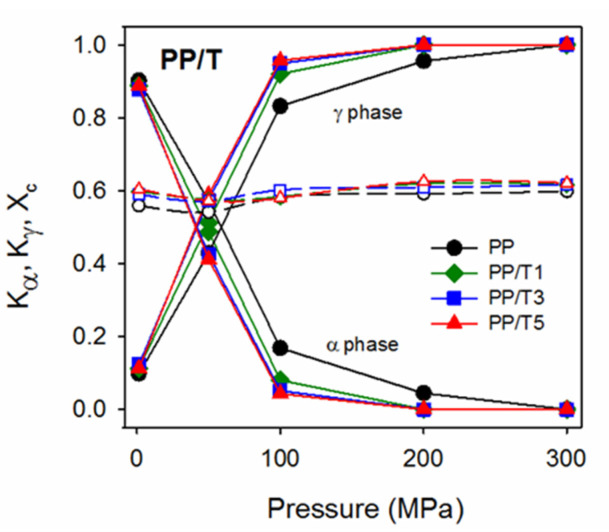
Phase contents in crystalline phase, K_α_ and K_γ_ (solid lines, filled symbols), and crystallinity degree, X_c_ (dashed lines, empty symbols), of neat PP and PP/T nanocomposites crystallized during cooling under various pressures.

Determination of the crystallographic modification of the crystals formed during cooling under various pressures was possible by WAXD. The analysis of the phase contents can take advantage of intensities of the (130)_α_ peak typical of the α-phase and (117)_γ_ peak characteristic of the γ-modification. Figure 6 shows the evolution of these peaks in diffractograms of PP and PP/T5 with increasing crystallization pressure. The diffractograms of PP and PP/T5 are presented in Figure S1 in Supplementary Information (SI) together with the diffractograms of PP/T1 and PP/T3, which were similar to that of PP/T5. Under 1.3 MPa all the materials crystallized in the predominant α-form, as can be judged based on the pronounced (130)_α_ peak and only a trace of the (117)_γ_ peak. With increasing pressure, the (130)_α_ peak decreased; whereas the (117)_γ_ peak increased in the diffractograms. In the diffractograms of PP crystallized under 200 MPa, the (130)_α_ peak became very weak; whereas it entirely vanished in the diffractograms of PP/T crystallized under the same pressure. In the diffractograms of all the materials crystallized under 300 MPa, this peak was absent evidencing the crystallization in the γ-form. It is worth mentioning that a small peak near 2θ of 18.3°, originating from the PTFE (100) crystallographic plane of hexagonal phase IV [48], appeared in the diffractograms of PP/T3 and PP/T5. Figure 7 shows the crystallographic form contents, K_α_ and K_γ_, and also crystallinity degree, X_c_, in the crystallized materials, determined based on the diffractograms. This confirms that under 1.3 MPa, PP and PP/T crystallized predominantly in the α-form, with a small γ-content in the crystalline phase. K_γ_ increased with increasing pressure, and in the 50–200 MPa pressure range it was higher in PP/T than in neat PP. At 200 MPa; the K_γ_ of PP was about 0.96; whereas it was equal to 1 for all PP/T nanocomposites. Under 300 MPa all the materials crystallized in the pure γ-form. It is worth mentioning that K_γ_ values of PP/T slightly increased with increasing PTFE content, especially in the pressure range of 50–100 MPa.

The increase of the γ-content with increasing pressure resulted from the broadening of the temperature range of the γ-phase formation [29]. Under 50–100 MPa the crystallization in PP and PP/T was not completed in the γ-domain and continued in the lower temperature range in the α-form. Under 200–300 MPa the crystallization occurred entirely or almost entirely in the γ-domain. The nucleating activity of PTFE, causing crystallization in a higher temperature range from more numerous nucleation sites, allowed reaching a higher crystallinity level in the γ-domain, increasing therefore the γ-content under elevated pressure, up to 200 MPa. The X_c_ of the PP matrix of all the materials, determined based on the diffractograms, was near 60 wt.% and weakly depended on the pressure and PTFE content.

**Figure 8:**
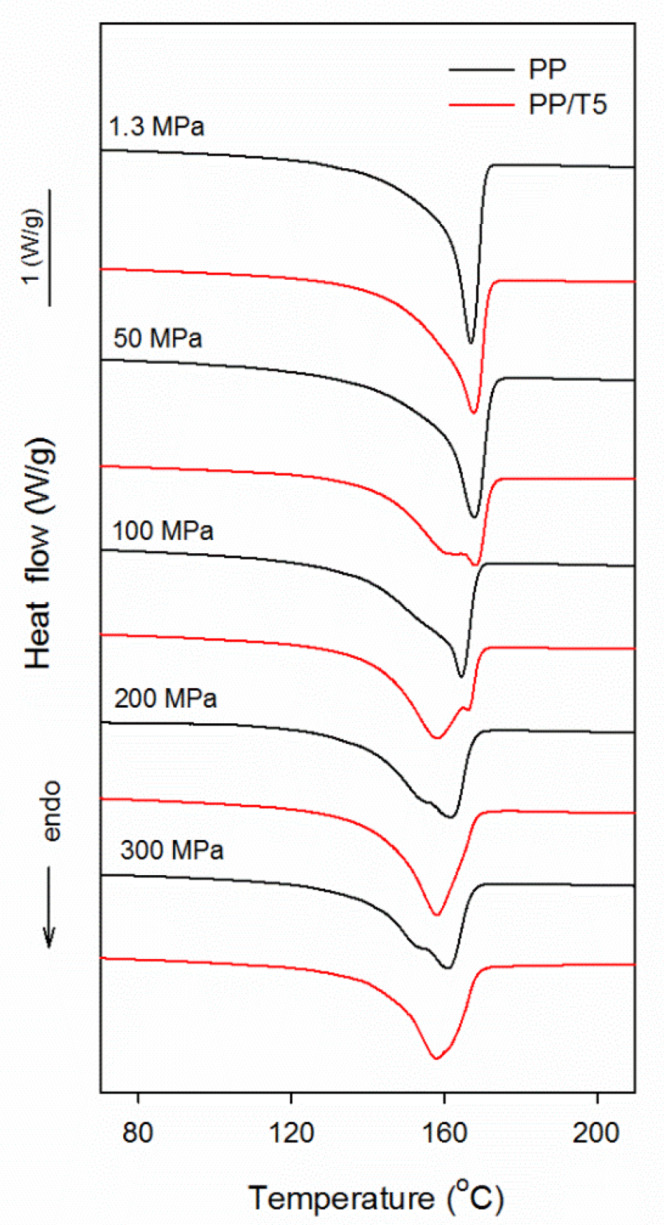
DSC heating thermograms of neat PP and PP/T5 nanocomposites crystallized during cooling under various pressures, recorded at 10 °C/min.

DSC heating thermograms of PP and PP/T5 recorded at 10 °C/min are collected in Figure 8. The thermograms of PP/T1 and PP/T3 were very similar to those of PP/T5, and are shown in Figure S2 in SI. The thermograms evidence the evolution, from melting of the predominant α-form crystallized under 1.3 MPa, to melting of the pure γ-modification crystallized under high pressure. The thermograms of PP crystallized under 1.3 MPa and 50 MPa were featured with single melting peaks, with melting peak temperatures (T_m_) of 167–168 °C. With increasing crystallization pressure, T_m_ decreased to 161 °C at 300 MPa, and the low temperature shoulders of the peaks grew and developed into additional peaks at 200 and 300 MPa. The thermograms of PP/T crystalized under 1.3 MPa were similar to that of PP, featured with single melting peaks with T_m_ of 168–169 °C, but with increasing pressure T_m_ decreased even more, to 157–158 °C. The melting endotherms of PP/T crystallized under 50 MPa exhibited additional low temperature peaks, which developed into the main peaks at 100 MPa, while the high temperature parts of the endotherms were reduced to small peaks or peak shoulders. The high-temperature shoulders of the melting peaks were also visible on the thermograms of PP/T1 crystallized under 200 and 300 MPa, but on the thermograms of PP/T3 and PP/T5 crystallized under the same conditions only weak traces of such shoulders remained.

The build-up of the low temperature peaks or shoulders and their evolution into the main melting peaks correlated with the increase of the γ-phase content with increasing pressure. The T_m_ of the γ-polymorph is below that of the α-modification, and during heating at 10 °C/min the γ-form melts, rather than transforms into the α-form [29]. It should be noted that the materials crystallized during cooling under 200 and 300 MPa contained pure or almost pure γ-form. Therefore, the double melting behavior or the presence of melting peak shoulders in thermograms of these materials most probably resulted from reorganization processes in the less stable γ-phase, formed during cooling at lower temperatures. These processes seem to be the strongest in neat PP and the weakest in PP/T5, which correlates with their T_c_ values.

In parallel to T_m_, the melting enthalpy of PP in the materials (calculated per PP content) also diminished with increasing crystallization pressure, from 102–107 J/g at 1.3 MPa, to 88–92 J/g at 300 MPa. This is because of the lower heat of fusion of the γ-modification, 190 J/g, than that of the α-modification, 209 J/g [29]. These values correspond to the similar X_c_ values of 49–51% and 46–48%, reached under 1.3 and 300 MPa, respectively. It should be noted that the X_c_ of PP/T, especially PP/T5 and PP/T3, was slightly higher than that of neat PP, due to the higher T_c_. In ref. [29] lower values of heat of fusion of the α- and γ-polymorphs were also considered, 167 J/g and 150 J/g, respectively. The X_c_ calculated assuming these values was approx. 60%, which agrees with the X_c_ based on WAXD results, plotted in Figure 7.

Previously [45], we studied the ability of 1,3:2,4-bis(3,4-dimethylbenzylidene)sorbitol (DMDBS) to nucleate iPP crystallization under elevated pressure. Others [49] observed that in the DMDBS concentration range of 0.2–1 wt.%, the molten iPP and the additive formed a homogeneous liquid, and that upon cooling the DMDBS crystallized prior to iPP, without a preceding separation into two liquids, and provided active nucleation sites for iPP crystals. It was demonstrated [45] that at least 0.4 wt.% of DMDBS was necessary to strongly enhance the nucleation of iPP under a pressure of 100 MPa or higher. In contrast, the fibrillated PTFE was present in the PP/T nanocomposites in the form of nanofibers, predominantly with diameters of 200 nm. However, the T_c_s of PP/T, even PP/T1, were similar to those of iPP with 0.4–1 wt.% of DMDBS. The neat iPP used in [45] crystallized with T_c_s lower by few degrees than the neat PP in the present study, due to the less intense nucleation. However, it is obvious that in iPP with DMDBS, the nucleation occurred on the nucleant, whereas in PP/T, it was on the fibrillated PTFE. Similar values of T_c_ indicate the strong ability of the fibrillated PTFE to nucleate crystallization of iPP during cooling in a broad pressure range. As a consequence, the γ-contents in iPP with 0.4–1 wt.% of DMDBS and in PP/T crystallized under elevated pressure were similar, with K_γ_ values of 0.58–0.6 and 0.51–0.59, respectively, under 50 MPa, whereas 0.92–0.95 and 0.92–0.96, respectively, under 100 MPa. Under 200 and 300 MPa both types of materials crystallized in the pure γ-form.

The obtained results evidenced the nucleation activity of fibrillated PTFE in PP in the entire pressure range studied, up to 300 MPa. According to the epitaxy theory [50], the heterogeneous nucleation of the α-phase of iPP mainly involves the (010)_α_ plane, and occurs on substrates matching a periodicity of 0.42, 0.5, or 0.66 nm in this plane. This epitaxy also applies for the γ-form involving the equivalent (001)_γ_ plane [50]. However, PTFE nucleates the iPP α-form through the epitaxy related to the (110)_α_ plane, which requires matching a periodicity of 0.55–0.56 nm [51]. This epitaxy does not apply for the γ-form. However, γ-lamellae also grow on α-crystals through conventional epitaxy of the γ-form on the (010)_α_ planes [37]. Thus, a possible mechanism leading to the nucleation of the γ-form under high pressure is the nucleation of the α-crystals, serving as seeds for the γ-lamellae. Although in the γ-domain the Gibbs free energy of the γ-form is lower than that of the α-form [29], the formation of a very small amount of α-phase serving as seeds for the γ-phase is still possible, and was observed by electron microscopy, even if not detected by WAXD [39,40]. Recently, we found [43] that this mechanism was predominant in nucleated iPP under high pressure. This can also explain the nucleation of PP in PP/T on the fibrillated PTFE.

## 4. Conclusions

Nanocomposites of PP with 1–5 wt.% of fibrillated PTFE (PP/T) were prepared by mixing PTFE powder with PP in the molten state. The fibrillation of PTFE in the materials was confirmed by SEM. Nonisothermal crystallization of the PP/T during cooling in a broad pressure range, up to 300 MPa, and the structure formed during the crystallization, were studied. Neat PP, without PTFE, was also analyzed, for comparison. The crystallization peak temperatures, T_c_s, of PP and PP/T during cooling increased with increasing pressure. The γ-phase content of crystallographic form in the crystalline phase, K_γ_, also increased, from about 0.1 at 1.3 MPa to 1.0 at 300 MPa. The T_c_s of the nanocomposites, especially PP/T3 and PP/T5, significantly exceeded those of neat PP, by up to 13 °C, depending on the pressure and PTFE content, due to the nucleating activity of the PTFE fibrils. The nucleating activity of the fibrils also resulted in a strong reduction of sizes of polycrystalline aggregates. The increase of T_c_ and decrease in size of the aggregates evidenced the ability of the fibrillated PTFE to efficiently nucleate crystallization of iPP in the γ-form under elevated pressure. This also enabled to achieve higher crystallinity level in the γ-domain, before the temperature decreased to the α-domain, and hence to increase the γ-content in PP/T, as compared to neat PP, crystallized during cooling under elevated pressure, up to 200 MPa, especially under 50–100 MPa. The obtained results indicate the significant influence of fibrillated PTFE on the crystallization and structure developed in PP under elevated pressure, which is of importance for processing, e.g., by injection molding.

## Data Availability

The data presented in this study are available on request from the corresponding author.

## Supplementary Materials

The following are available online at https://www.mdpi.com/article/10.3390/polym14010088/s1, Figure S1: WAXD diffractograms of PP and PP/T crystallized during cooling under various pressures., Figure S2: DSC heating thermograms of PP/T1 and PP/T3 nanocomposites crystallized during cooling under various pressures, recorded at 10 °C/min.
