# Supplementary material for: Crystallization of Isotactic Polypropylene Nanocomposites with Fibrillated Poly(tetrafluoroethylene) under Elevated Pressure
Source: Polymers (Basel). 2021 Dec 27;14(1):88. doi: 10.3390/polym14010088 (PMC8747199; doi:10.3390/polym14010088)
Supplement: Supplementary file 1 [file polymers-14-00088-s001.zip › polymers-1501621-supplementary.pdf]

Supplementary Information

Crystallization of isotactic polypropylene nanocomposites with fibrillated poly(tetrafluoroethylene) under elevated pressure

Przemyslaw Sowinski *, Sivanjineyulu Veluri, Ewa Piorkowska *

Centre of Molecular and Macromolecular Studies, Polish Academy of Sciences, Sienkiewicza 112, 90-363 Lodz, Poland; sveluri@cbmm.lodz.pl

* *Correspondence: przem_so@cbmm.lodz.pl (P.S.); epiorkow@cbmm.lodz.pl (E.P.); Tel.: +48-42-6803316 (P.S.)*

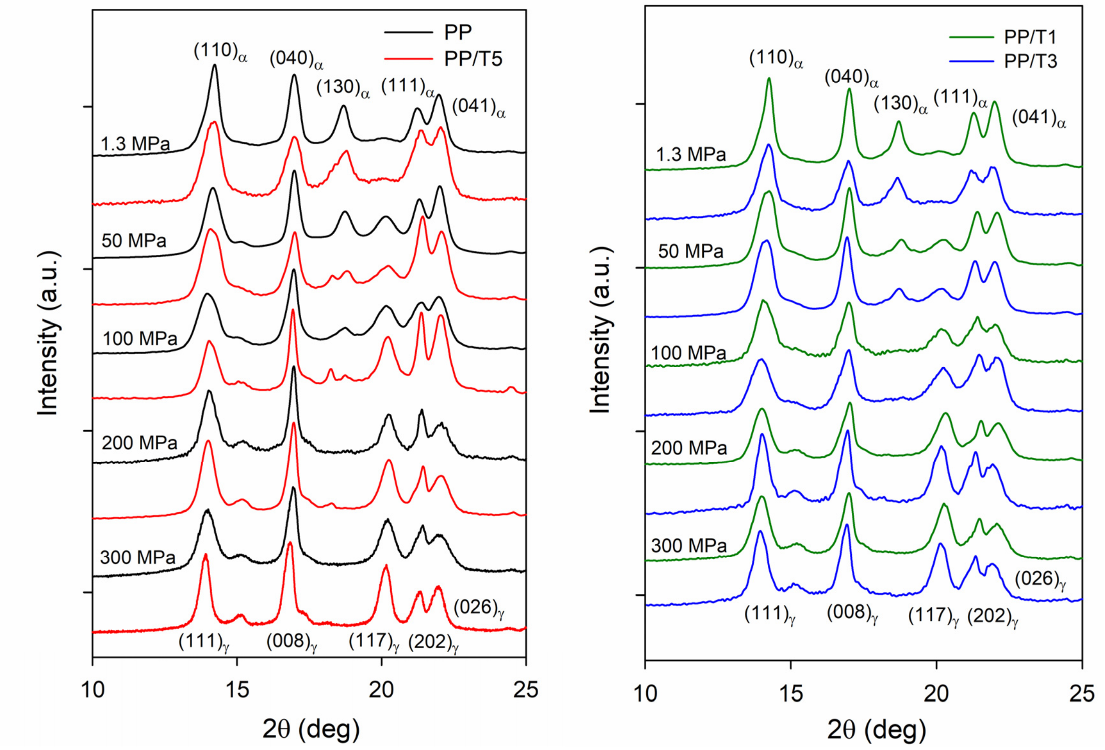

Figure S1. WAXD diffractograms of PP and PP/T nanocomposites crystallized during cooling under various pressures.

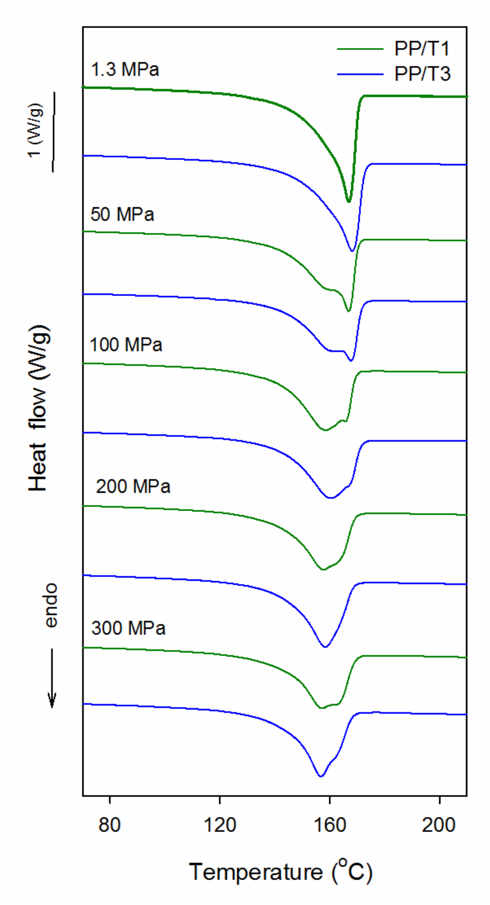

Figure S2. DSC heating thermograms of PP/T1 and PP/T3 nanocomposites crystallized during cooling under various pressures, recorded at 10 °C/min.
